# Behavioral traits and territoriality in the symbiotic scaleworm *Ophthalmonoe pettiboneae*

**DOI:** 10.1038/s41598-021-91810-2

**Published:** 2021-06-11

**Authors:** Temir A. Britayev, Daniel Martin

**Affiliations:** 1 A. N. Severtzov Institute of Ecology and Evolution (RAS), Moscow, Russia; 2 Centre D’Estudis Avançats de Blanes (CEAB-CSIC), Blanes (Girona), Catalunya Spain

**Keywords:** Animal behaviour, Behavioural ecology

## Abstract

Among marine invertebrates, polychaete worms form symbiotic associations showing a wide variety of host use patterns. Most commonly, they live solitary on hosts, likely resulting from territorial behavior, yet little is known of the precise nature of the involved interactions. Based on field and laboratory observations, we described the symbiotic association between *Ophthalmonoe pettibonae* and *Chaetopterus* cf. *appendiculatus* from Nhatrang Bay (Vietnam). Then, by experimentally manipulating the competitor-to-resource ratio, we analyzed symbiont behavior and we assessed whether the 1:1 uniform distribution observed in nature could be driven by agonistic territorial behavior. Hosts and symbiont populations had low densities, lacked size relationships and showed higher prevalence when denser. Symbiont behavior included territoriality, expressed through conspecific recognition and intraspecific aggressive interactions (pursuit and escaping, hiding, choosing position, aggressive fighting, and targeting a specific bite zone). Our experiments proved that territoriality led to host monopolization by a single symbiont, provided the first empirical evidence that symbiont body injuries were caused during territorial contests, and allowed us to first suggest that a marine symbiotic invertebrate may control a territory extending beyond its host, even including neighboring hosts. Overall, this is the first report of such a complex symbiotic behavior for an annelid polychaete.

## Introduction

Many organisms show resource-guarding behaviors to control resources such as shelter, food, offspring or mating associates^1,2^. Predictive models show a parallel evolution of resource-guarding behavior when possible intruders are in low abundance, but also when resources are aggregated and/or sparse^3^. External factors controlling behavioral expressions are well known for terrestrial and marine taxa, including fish^3^, social shrimps^4^ or symbiotic crabs^1,5,6^. Resource scarcity has traditionally been considered among the main ecological factors leading to the establishment of resource monopolization and guarding behaviors in invertebrates, including territoriality^7^. Many animals typically have specific home range areas in which individuals perform most daily activities, but some also defend territories^8–10^. Territories are fixed areas used exclusively by one or more individuals, from which they attempt to exclude potential competitors via agonistic or aggressive behavior^7^. This behavior generally involves intra- and interspecific contests^11^ and has more commonly been studied in vertebrate systems, but also documented in marine invertebrates^12–14^.

Symbiotic organisms develop persistent, intimate associations with their hosts, frequently with a high degree of specificity, to the extent that one of the associates can no longer survive as free-living^15^. For marine symbiotic fish and invertebrates, hosts are vital resources providing protection from predators, competition and environmental stress, food, and mating and larvae hatching spaces. Therefore, the necessity of preserving such a resource predicts widespread territoriality among symbionts in the marine environment^16–20^.

In symbiotic marine invertebrates, host bodies or structures associated with them (e.g., shells, tubes, burrows) are often the territory being defended^16,18,21^. Defending small, simple and sparsely distributed hosts requires less investment of time and energy^5,22^, while the energetics of defending complex and densely distributed hosts becomes too high, forcing symbionts to share hosts with conspecifics^23,24^. However, it is even more energetically expensive when one individual or one pair of symbionts must control more than one host, which is rare in marine environments. Such a behavior was reported in anemonefish^25–27^, but it is unknown in marine invertebrates. Host-use patterns vary widely depending on the ecology of hosts and symbionts, as reported in marine decapods^14,28–31^. Some symbionts show large structured aggregations^28^, while others live in heterosexual pairs^14,32–34^ or solitary^5,35,36^. However, the role of territoriality in establishing these patterns remains poorly understood.

Among marine invertebrates, the ubiquitous annelid polychaetes include more than 600 species living in symbiosis with representatives from all known major groups of marine animals, most of them being strictly ‘monoxenous’ (i.e. associated with a single host species), but showing a variety of spacing systems and intraspecific association patterns on/in their hosts^18,37^. Serpulids and antonbruunids show dense unstructured aggregations and probably lack territorial behavior^38–40^, while chrysopetalids and polynoids may occur as heterosexual pairs^41,42^. Most commonly, however, symbiotic polychaetes live solitary on their hosts^18,37^. Having an aggressive territorial behavior has been postulated for hesionids^43,44^, nereidids^45^ and, particularly, polynoids^46–48^. However, few studies described or performed experiments on territorial behaviors and their relationships with patterns of host use^12,45,49–51^. With a few exceptions^43^, territoriality was indirectly inferred from uniform 1:1 or 2 (male/female):1 distributions^18,37^ or the presence of injuries^43,46,47,49,52^. However, direct cause/effect relationships have not been reported to date, except to show that intraspecific aggressiveness occurred only in the presence of hosts^44^.

Territorial behavior in polychaetes (either free-living or symbiotic) appeared to be less complex than in decapods^12,53–55^. *Arctonoe pulchra* (Johnson, 1897)^56^, the best studied symbiotic polychaete in this matter, only showed two behavioral actions during aggressive interactions (i.e., bouncing on contact and biting)^12^. This pales compared with the more complex sequence of aggressive displays and fighting of the crabs *Trapezia intermedia* Miers, 1886^57^ and *T. digitalis* Latreille, 1828^14,58^.

The symbiotic scaleworm *Ophthalmonoe pettiboneae* Petersen and Britayev, 1997^59^ (Annelida: Polynoidae) lives exclusively in association with the tubeworm *Chaetopterus* cf. *appendiculatus* Grube, 1874^60^ (Annelida: Chaetopteridae)^46,59,61^ (Supplementary material S1). Its strict 1:1 uniform distribution and the presence of body injuries^61^ suggest territorial behavior^1,3,21^.

In this paper, we are describing the characteristics of this association (i.e., field density of host populations, prevalence, and host/symbiont size relationships) and the behavior of the symbiont (based on direct observations in experimental conditions). Moreover, we are using laboratory experiments to assess host/symbiont and symbiont/symbiont relationships. We are also experimentally manipulating competitor-to-resource ratio (CRR = number of potential competitors divided by number of resource units in a population at a given time)^62^, aiming at determining whether the 1:1 uniform distribution observed in nature is driven by intraspecific interactions and agonistic territorial behavior. Based on preliminary field observations on host use patterns^46^ and on previous experiments manipulating competitor-to-resource ratio in symbiotic scaleworms^12^ and crabs^63^, we expected that both a ratio equal to two (two symbionts/one host) and equal to one (two symbionts/two hosts) would led to the entrainment of all available hosts by only one symbiont.

## Results

### Characteristics of the association

We collected 84 hosts (22 in Mun Island, 23 in Mot Island, 19 in Point Nam and 20 Dam Bay) and 53 symbionts (10 in Mun Island, 16 in Mot Island, 12 in Point Nam and 15 Dam Bay). The overall prevalence was 63.1% and the intensity of one symbiont per host. Only in one case, the scaleworm shared the host with a pair (male and female) of the symbiotic crab *Eulenaios cometes* (Walker, 1887)^64^.

**Figure 1:**
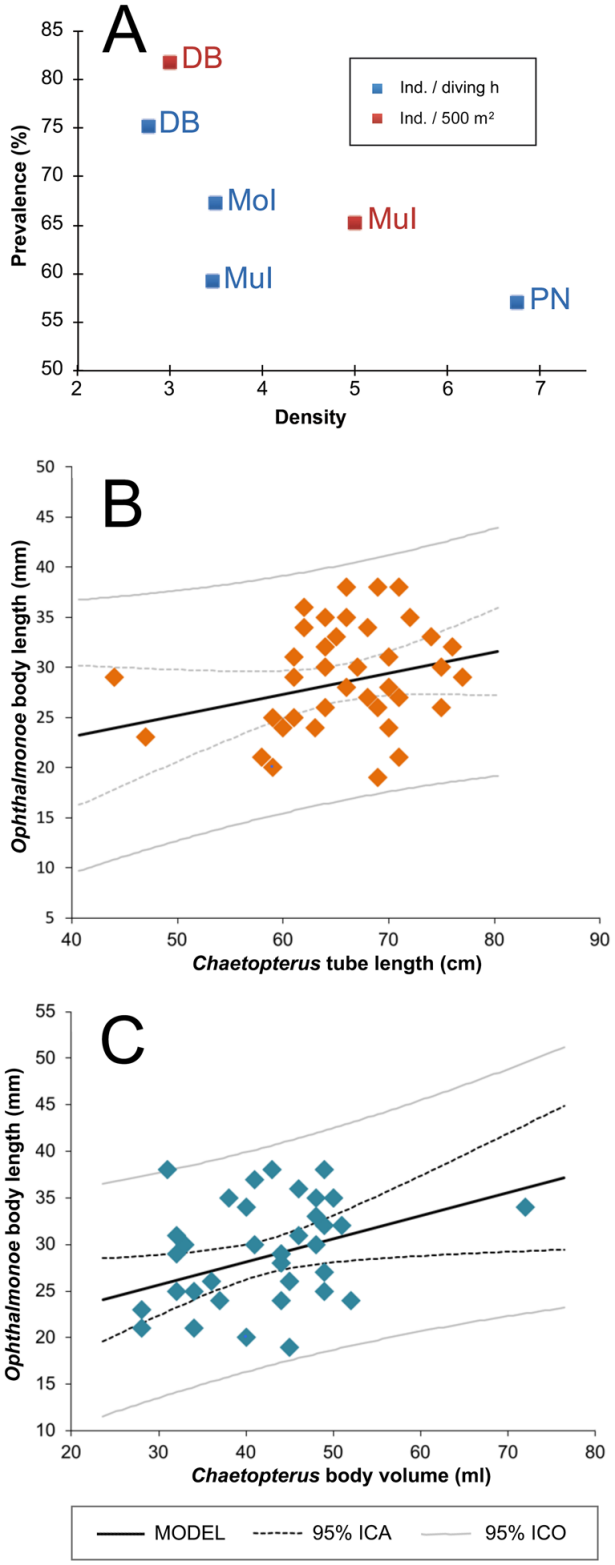
(**A**) Relationships between host density and prevalence. (**B**) Host tube length versus symbiont body length. (**C**) Host body volume versus symbiont body length. ICA: Interval of Confidence over the Average; ICO. Interval of Confidence over the observed values; DB: Dam Bay; MuI: Mun Island; MoI: Mot Island; PN: Point Nam, Tre Island. Plots created with the XLSTAT software version 18.03 (https://www.xlstat.com).

Host densities were low, from 3 (Dam Bay) to 7 (Point Nam) ind.·diving h^−1^ and from 3 (Dam Bay) to 5 (Mun Island) ind. · 500 m^−2^, clearly indicating a patchy distribution. The prevalence ranged from 75% (Dam Bay) to 57% (Point Nam) and from 81% (Dam Bay) to < 66% (Mun Island), decreasing with increasing density (Fig. 1A).

All hosts were at a distance of tens of meters of each other except three cases where two hosts were at 1 m or closer. In two of them, there was only one symbiont inside one of the hosts. In the other, both hosts harbored symbionts, one being seriously damaged (it was an anterior fragment with nearly 10 segments lacking elytra and several parapodia, without traces of regeneration).

The length relationship between symbiont body (BL) and host tube (TL) was non-significant (BL = 14.657 + 0.210 · TL, R^2^ = 0.062, *p* = 0.123; Fig. 1B), while symbiont length slightly increased together with the increasing host body volume (BV), although the variance explained was very low (BL = 18.183 + 0.284 · BV, R^2^ = 0.122, *p* = 0.027; Fig. 1C).

### Host/symbiont interactions

#### Host entering behavior

**Figure 2:**
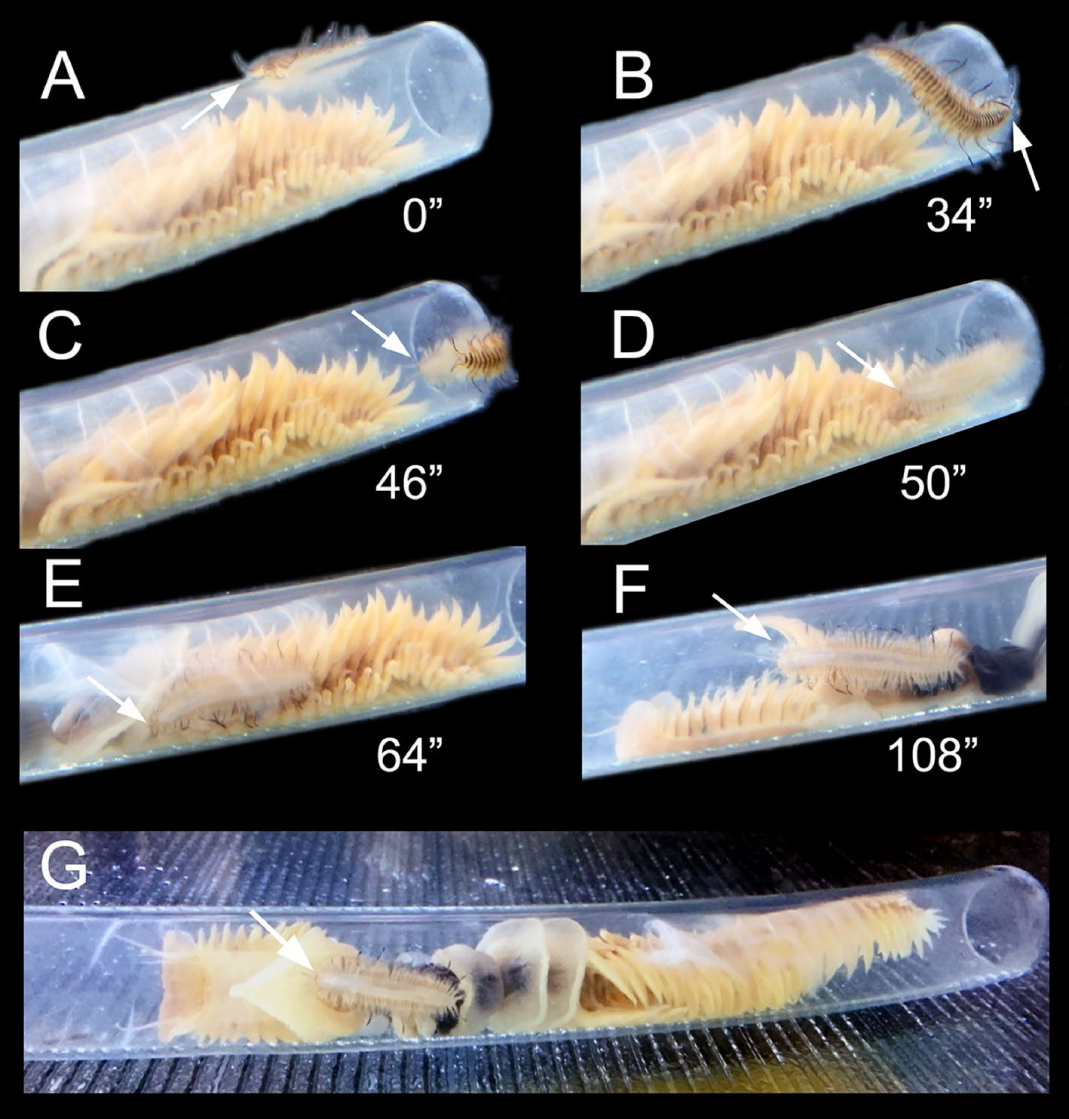
*Ophthalmonoe pettiboneae* host entering sequence, see Supplementary material S3. (**A**) Crawling outside the tube. (**B**) Approaching the exhalant siphon. (**C**) Entering through the exhalant siphon. (**D**) Already inside the tube. (**E**) Crawling inside the tube. (**F**) Reaching the typical position inside the tube. (**G**) Dorsal view of an example of the typical position inside the tube. White arrows indicate the symbiont head. Time course indicated in seconds.

The symbionts always entered the host tube through the exhalant syphon (Host/Symbiont experiment, 5/5 replicates; Symbiont/Symbiont experiment and Manipulating CRR experiment, all observations) (Fig. 2, Supplementary material S3). After detecting the host, the symbiont lied for a short time on the tube external surface, close to the exhalant opening, with its head towards it (Fig. 2A). Then, it entered the tube and moved rapidly between the host body and the inner tube wall, with its ventral side oriented towards the wall (Fig. 2B–D), and easily overpassed the host region C (Fig. 2E) and the pumping segments to reach a position near region A (Fig. 2F). While inside the tube, the symbiont also easily moved from left to right host sides, either crossing between the tube and the host dorsal side almost avoiding touching the host, or between the tube and the host ventral side gently forcing the host to move up.

#### Symbiont location

Soon after entering the tube, the symbiont lied between the end of the host region A and the middle of region B (Host/Symbiont experiment, 5/5 observations; Manipulating CRR experiment, 18/28 observations; Figs. 2G, Supplementary materials S3 and S4), where the host builds its filtration net (Supplementary material S1). In most cases, the symbiont had its head directed towards the host head and the inhalant siphon, with its ventral side in contact with the host tube and the dorsal side oriented towards the host body, without altering host venting (Supplementary material S4). Thus, we consider this as its typical position. Symbionts held this position for long time, moving rarely except during host reversal (Supplementary material S5 and S12), neither attempting to bite the host, nor stealing food from the host filtration system. Only once we observed a symbiont snatching a small food particle from the water pumped inside the tube.

#### Host/symbiont synchronous behavior

Host reversal was always mirrored by the symbiont (Supplementary material S5 and S12). The scaleworm first moved backwards following the initial host movement. When host region A reversed, and before starting reversing region B, the symbiont quickly changed its orientation to direct its head towards the new position of the host head. The symbiont remained near region A until the host fully adopted its new orientation and then moved slowly to adopt the typical position.

### Symbiont/symbiont interactions

#### Interactions inside host tube

##### Resident symbiont larger than intruder

**Figure 3:**
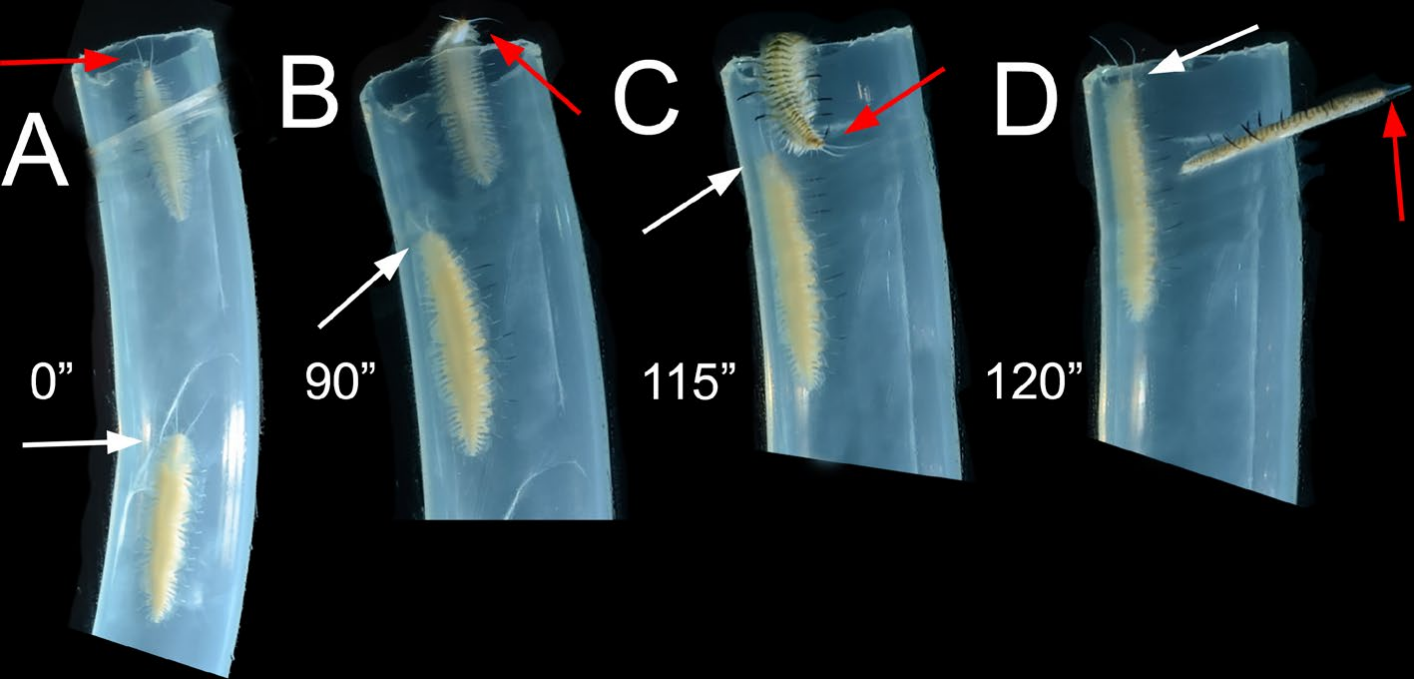
Sequence of a territorial interaction, with resident *Ophthalmonoe pettiboneae* intimidating an intruder, leading to the latter leaving the tube without being attacked by the former. (**A**) Progressive approach of the resident towards the intruder, with no reaction from the intruder. (**B,C**) Resident still approaching and intruder with part of its body outside the tube. (**D**) Resident at the tube opening and intruder swimming outside the tube. White arrows indicate the resident’s head; red arrows indicate the intruder’s head. Time course indicated in seconds.

In one Symbiont/Symbiont experiment and one Manipulating CRR experiment replicates, the intruder entered the occupied host and was detected by the resident, which started moving slowly towards it. As soon as the intruder perceived this resident movement, it turned around and quickly abandoned the host (Fig. 3A–D). The attempt ended without any other interaction.

**Figure 4:**
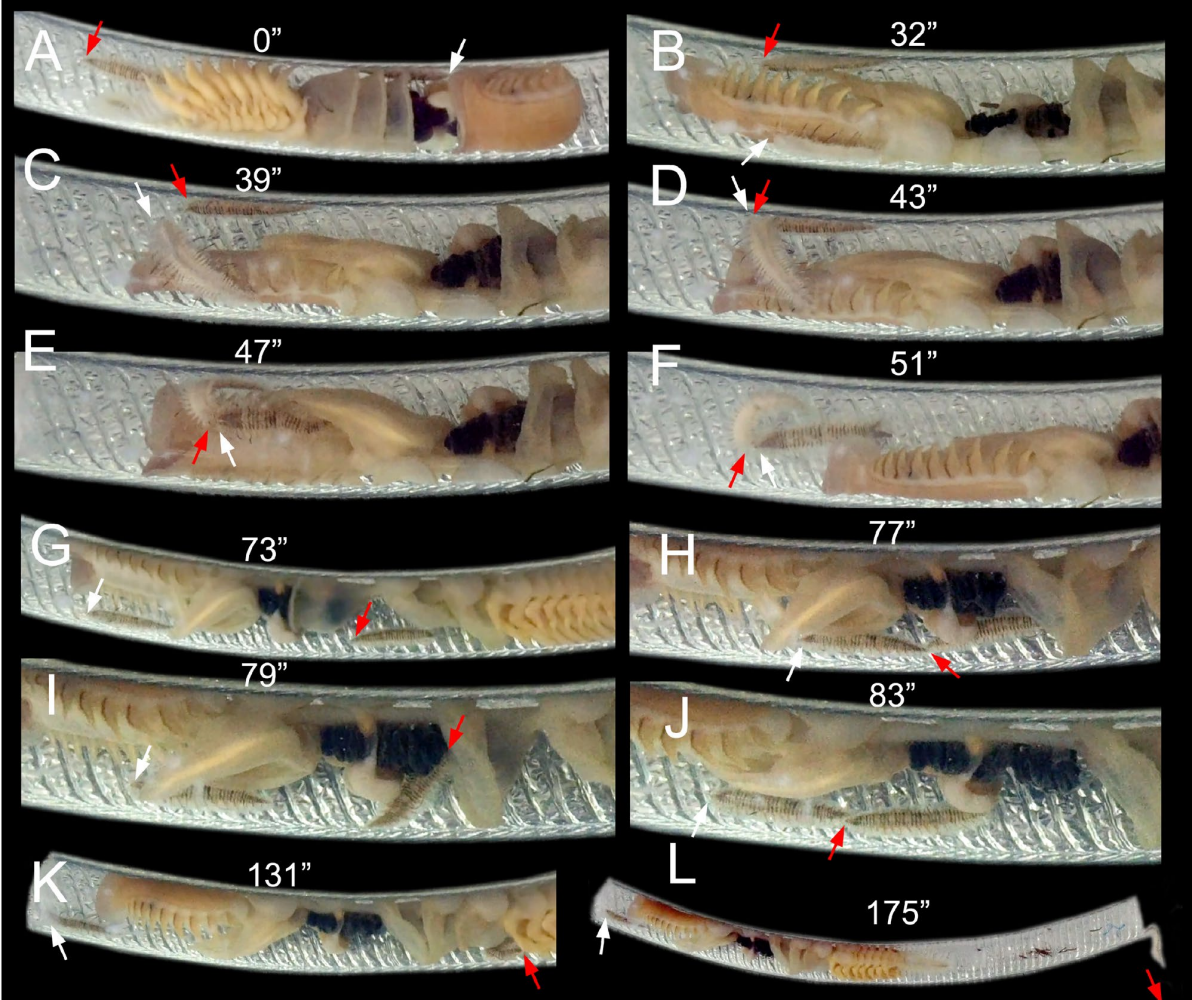
Sequence of a resident *Ophthalmonoe pettiboneae* attacking an intruder; see Supplementary material S6. The intruder is always in the same position on the inner tube wall, except after the attack. (**A**) Resident in its typical position, host starting to reverse its body. (**B**) Resident hidden behind host body, host fully reversed. (**C**) Resident closely approaching the intruder’s anterior end. (**D**) Resident starting the attack. (**E**) Resident curling with the intruder during the attack, host slowly retracting its body. (**F**) Resident still biting the intruder, both curling one over the other, host apparently giving them room to fight. (**G**) Resident near host’s anterior end, intruder around host’s mid-body. (**H**) Intruder closely approaching the resident’s posterior end. (**I**) No reaction from resident, but intruder suddenly reversing its body. (**J**) No reaction from resident, intruder again closely approaching the resident’s posterior end. (**K**) Resident slowly moving towards the inhalant siphon, intruder already oriented towards the exhalant siphon. (**L**) No reaction from resident, intruder almost already outside the tube. White arrows indicate resident’s head; red arrows indicate the intruder’s head. Time course indicated in seconds.

In other three Manipulating CRR experiment replicates, the intruder initially did not interact with the resident, and was not immediately detected. Both scaleworms remained in different parts of the tube for some time (Fig. 4A, Supplementary material S6). When the host started to reverse, the resident also reversed, almost immediately detected the intruder and reacted brusquely and very quickly (Fig. 4B, Supplementary material S6). The resident began advancing towards the intruder, jumping from one side of the inner tube to another and being not detected by the intruder due to the presence of the host body between them (Fig. 4C, Supplementary material S6). Perceiving the intruder from behind the host body, the resident launched a brisk attack, suddenly everting its pharynx and biting the intruder with its jaws (Fig. 4D, Supplementary material S6). Then, both worms curled one over the other for about 30–60 s (Fig. 4E–F, Supplementary material S6), ending with the resident extracting a piece of the intruder’s body.

A single attack was often enough to force the intruder to abandon the host, although it took some time to leave (i.e., about 2 min; Supplementary material S6). In some instances, it even approached the resident several times (Fig. 4G–J, Supplementary material S6). Such an approach triggered either no attacks (twice) or a second attack (once) from the resident. In both cases, the intruder finally moved fast towards the exhalant tube opening and quickly exited, swimming as far as allowed by the experimental aquaria (Fig. 4K–L, Supplementary material S6). In these conditions, we never observed more than two successive attacks.

##### Resident symbiont smaller than intruder

**Figure 5:**
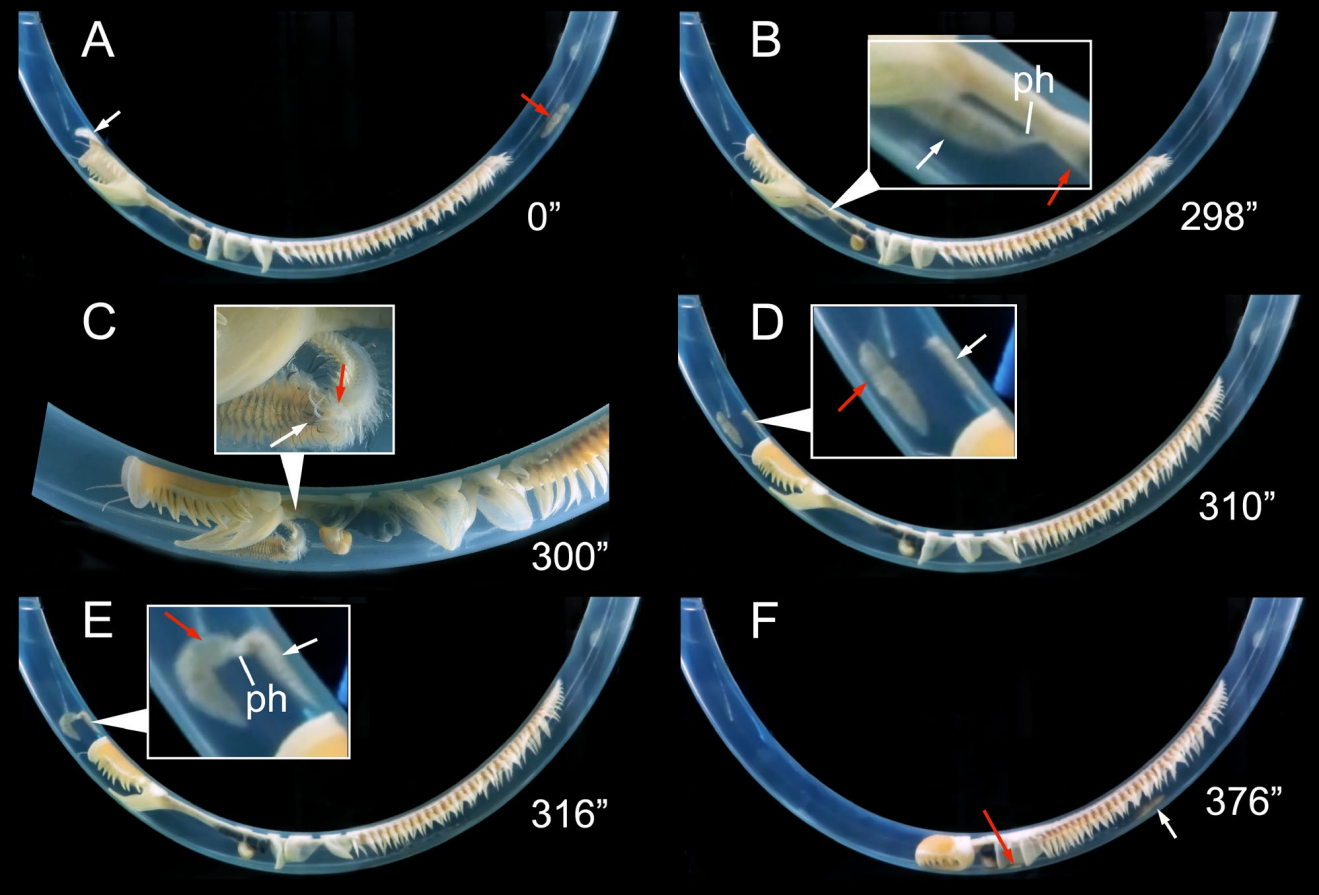
Sequence of two territorial attacks between a small resident and a large intruder *Ophthalmonoe pettiboneae*; see Supplementary material S7. (**A–C**) Small resident attacking the large intruder: (**A**) resident near the host head, intruder just entered the tube; (**B**) Resident just attacked the intruder; (**C**) resident and intruder curling one over the other during the attack. (**D–E**) Large intruder attacking and driving out the small resident: (**D**) intruder approaching the resident; (**E**) Intruder attacking the resident; (**F**) intruder in the typical position, resident moving, going towards the exhalant siphon to leave the tube. Ph: Pharynx. White arrows indicate the smallest resident; red arrows indicate the largest intruder. Time course indicated in seconds.

We observed the entrance of a larger intruder inside a tube occupied by a smaller resident initially triggering the resident’s attack (Fig. 5A–C, Supplementary material S7) and almost immediately being followed by an intruder’s attack (Fig. 5D–E, Supplementary material S7). However, the resident remained in the tube (Fig. 5F, Supplementary material S7) and only left it after a second attack by the intruder. In all observational and experimental set-ups (i.e., 22 replicates), this was the only case in which we observed three successive attacks, one performed by the resident and two by the intruder.

#### Interactions outside the host tube

**Figure 6:**
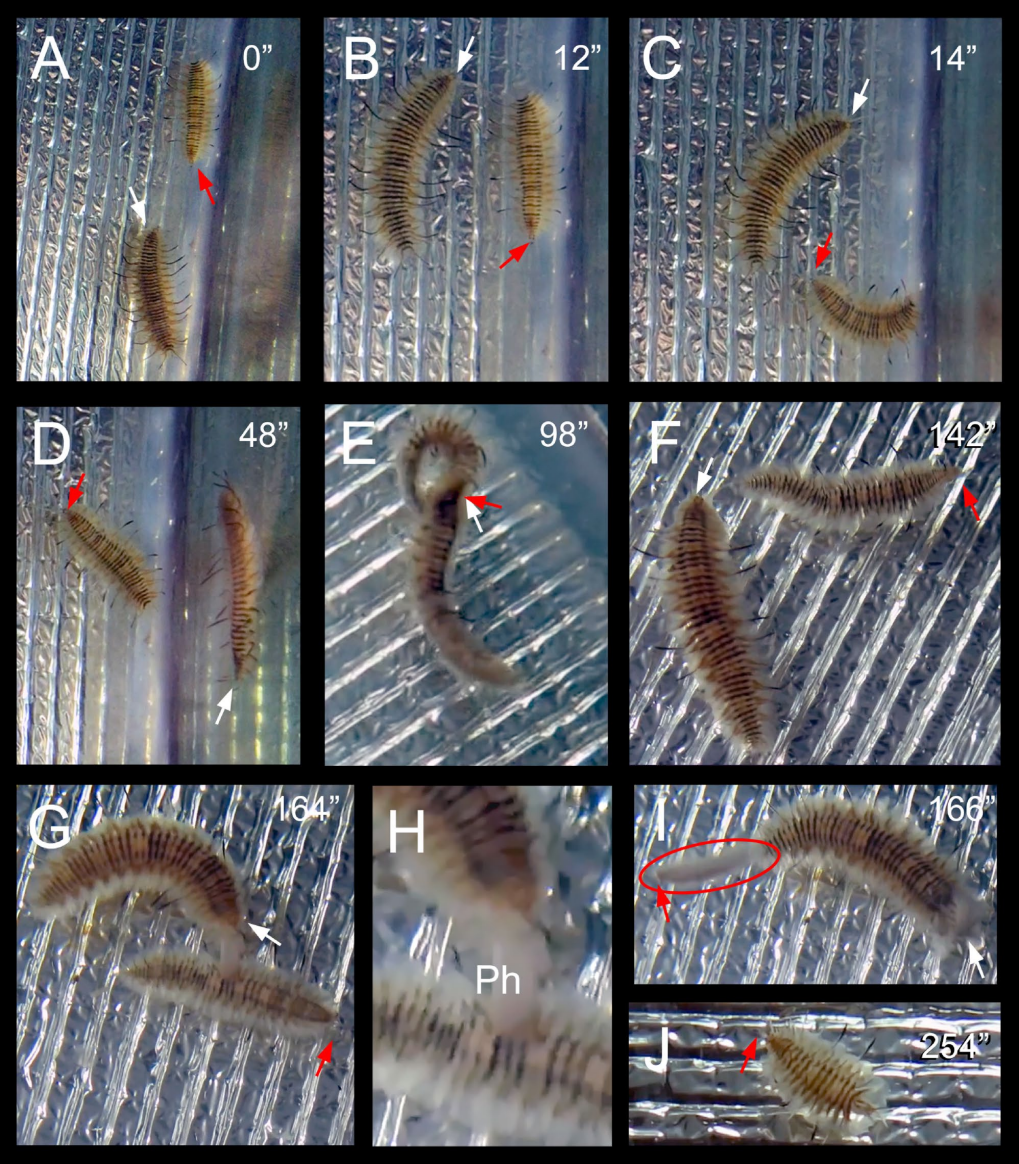
Sequence of two territorial attacks of a large against a small specimen of *Ophthalmonoe pettiboneae* outside the host tube, in which the smaller was finally torn in two; see Supplementary material S8. (**A**) Large scaleworm approaching the smaller. (**B**) Large scaleworm overpassing the smaller, the smaller moving to face the larger. (**C**) Large scaleworm after a sudden movement to find the adequate position for attack. (**E**) First attack. (**F**) Small scaleworm moving slowly, large scaleworm preparing a second attack. (**G**) Large scaleworm just everting its pharynx during the second attack. (**H**) Close view of the start of the second attack. (**I**) Anterior region of the small scaleworm (red oval) quickly moving out of the attack zone after being autotomized, posterior region still trapped by the pharynx of the large scaleworm. (**J**) Detail of the anterior fragment of the small symbiont after the attack. Ph: Pharynx. White arrows indicate the head of the large specimen; red arrows indicate the head of the small specimen. Time course indicated in seconds.

During the Manipulating CRR experiment, we observed four times one symbiont following the tracks (likely chemical) of another (Fig. 6A–D, Supplementary material S8). In two cases, the larger symbiont chased the smaller, approaching it from its posterior end, which probably prevented the smaller from immediately detecting the larger. Then, the larger moved fast to face the smaller (e.g., a slightly elevated position on the lateral aquarium wall), while the smaller reacted by orientating its anterior end towards the larger (likely a visually-mediated interaction). The situation either remained static for some time or the two symbionts stayed face to face and moved slowly. After a few seconds, the larger symbiont undertook the same brisk attack as inside the host tube (Fig. 6E, Supplementary material S9). In one case, this was enough to force the small symbiont to swim out. In the other case, the smaller symbiont crawled slowly without abandoning its position, which triggered a second attack that finally forced it to rapidly swim towards the other side of the experimental aquarium (Fig. 6F–I, Supplementary material S9). Either after one or two attacks, the larger symbiont crawled slowly to enter the host tube.

Smaller symbionts chasing larger ones did not attack them (two observations). Instead, they undertake escape reactions after approaching the large symbionts, moving away either very fast (Supplementary material S10) or slowly (Supplementary material S11).

### Manipulating competitor-to-resource ratio (CRR) experiment

**Figure 7:**
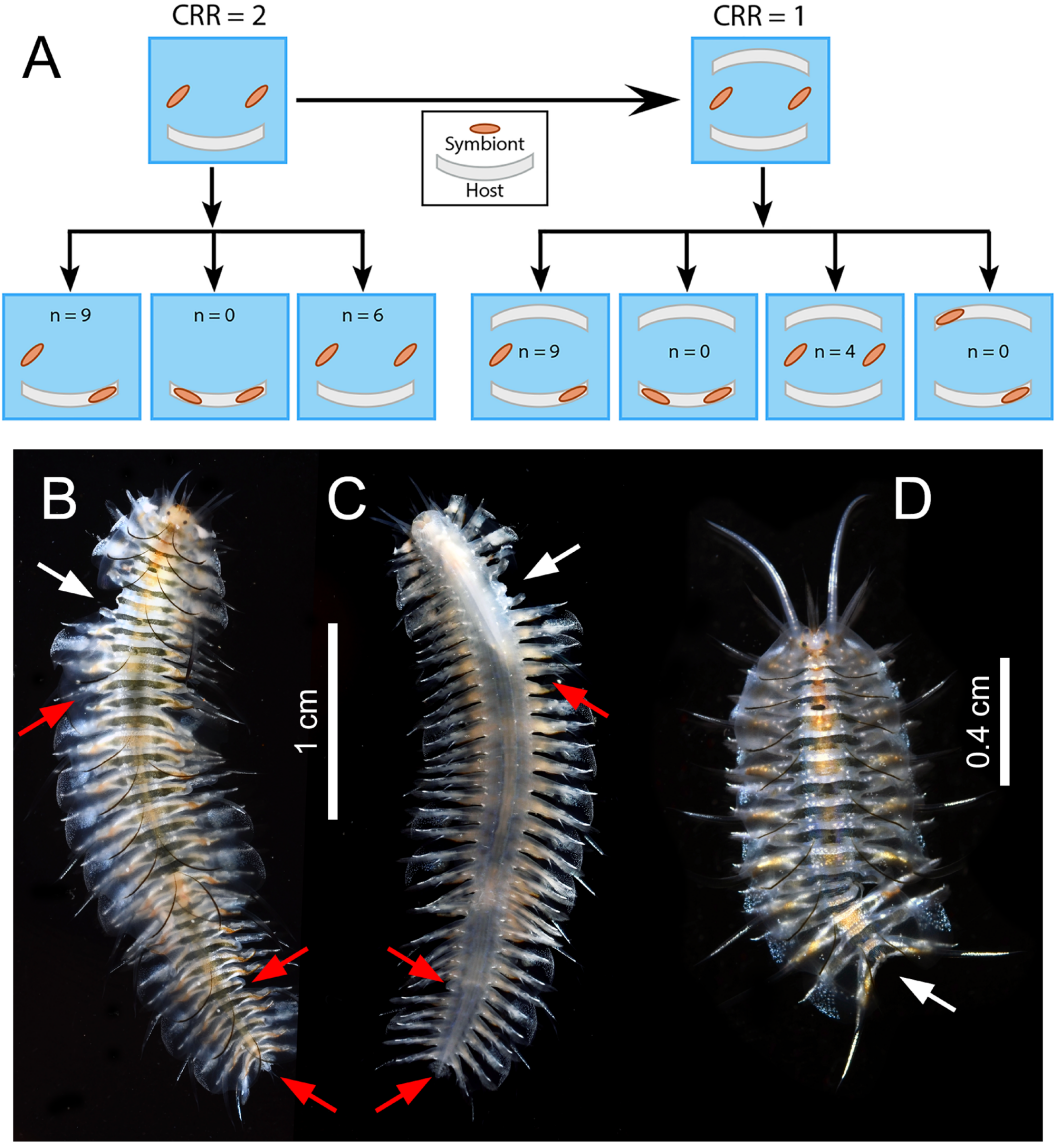
(**A**) Scheme of the treatment design in the manipulating competitor-to-resource ratio (CRR) experiment, showing the outcome of the two treatments. Dorsal (**B**) and ventral (**C**) views of an intruder *Ophthalmonoe pettiboneae* showing both recent (four left parapodia, two elytra) and old, regenerating (two left and one right parapodia, several posterior segments) injuries. (**D**) *Ophthalmonoe pettiboneae* with a posterior injury affecting ca. two body thirds; white arrow: recent injury; red arrow: old, regenerating injury.

In the CRR = 2 treatment, the number of hosts harboring a single symbiont versus no symbiont versus two symbionts differed significantly from each other (critical Chi-square = 7.8, *p* < 0.001). In nine replicates (60%) there was one symbiont inside the host and one outside (Fisher exact test, *p* = 0.05). In six replicates (40%) both symbionts were outside the host (Fisher exact test, *p* = 0.05). Cases of two symbionts sharing the same host were never recorded (Fig. 7A). During all replicates, we observed: (1) one symbiont chasing the other, twice outside and once inside the tube, (2) one independent attack inside the tube that occurred during daytime, and (3) two successive attacks outside the tube that occurred during nighttime.

In the CRR = 1 treatment, the number of hosts harboring a single symbiont versus no symbiont versus two symbionts also differed significantly from each other (critical Chi-square = 9.4, *p* < 0.001). The case with one symbiont inside the host and one outside was significantly more common (Fisher exact test, *p* = 0.05), although the prevalence was substantially reduced with respect to the CRR = 2 treatment (i.e., only nine hosts—35%—harbored a symbiont). The case with both symbionts outside the tube did not differ significantly, while cases where two symbionts shared a host and each host harbored one symbiont were never recorded (Fig. 7A). During all replicates, we observed: (1) twice, one symbiont chasing the other outside the host, ending with either one escape reaction and one attack, and (2) two independent attacks inside the host that occurred during daytime.

In both Manipulating CRR experiment treatments, large symbionts were significantly more frequent inside the hosts (and small symbionts outside) (Fisher test, *p* = 0.05), while the contrary (i.e., small inside and large outside) did not differ significantly (Table 1).

**Table 1:** Outcome of the two treatments of the manipulating competitor-to-resource ratio (CRR) experiment and results of the contingency analysis considering symbiont size.

	CRR = 2	CRR = 1
Large inside small outside	**7**	**6**
Small inside large outside	2	3
Two inside same tube	*0*	*0*
Two outside	**6**	4
Two inside different tubes	*–*	*0*

Bold: observed > theoretical; italics: observed < theoretical. H: host; S: symbiont.

### Presence of injuries

A 20% of the scaleworms collected in situ showed lateral injuries in different phases of regeneration, while only one showed a posterior (recent) injury. During our experiments, the symbionts suffered both types of injuries as a result of independent or successive attacks, (both inside and outside the host). Lateral ones were more frequent (85%, 11 of 13 injured individuals, Fig. 7B,C) and were often located in the pharyngeal area. In one case, an attack directed at the anterior body region of the opponent induced autotomizing a very long posterior section. However, there were no indications of cannibalism, as the bitten fragment remained in the aggressor jaws for some time but was finally rejected (Figs. 6G–J, 7D, Supplementary material S9). Recent injuries were found in 46% of the scaleworms used in the Manipulating CRR experiment (n = 28). One or both symbionts showed injuries in 77% of replicates at the end of the CRR = 1 treatment, which largely surpasses the fighting events registered during our direct observations. Both large and small scaleworms were injured (n = 13), and injuries were more common in small (69%) than in large (31%) symbionts.

## Discussion

### Territoriality and spatial distribution

The tropical soft-bottoms inhabited by *C.* cf. *appendiculatus* had a low structural complexity, thus offering few refuges from roving predators (authors’ personal observations). Accordingly, the very low abundance and wide dispersion of their populations did not favor the movement of symbionts from host to host. Conversely, defending the host may be favored by the U-shaped tube, the narrowness of the two siphons and the ca. 1:4 symbiont:host size ratio (i.e., 4 and 15 cm long, respectively)^46^, and should not require much investment in energy and time. As already proposed for symbiotic crustaceans^1,21^, these features predicted territorial behavior in *O. pettiboneae*.

Our results supported that *O. pettiboneae* was a strongly territorial, solitary species that monopolized and aggressively defended a host from conspecific attempts to enter its tube. All aggressive interactions led one symbiont, usually the smaller individual, to leave the host, which may or may not involve actual physical interactions. As a consequence, at the end of our experiments, all hosts were colonized by solitary symbionts, leading, as expected, to the establishment of a uniform 1:1 distribution. However, a second symbiont was not able to colonize a host, even when two were available.

### The territory in *Ophthalmonoe pettiboneae*

One may expect that a symbiont forced to leave a host (i.e., the loser in the intraspecific interactions) must seek shelter, preferably inside another host. Nevertheless, 40% of hosts remained unoccupied at the end of the CRR = 2 treatment, while a twofold decrease in the competitor-to-resource ratio did not lead the new hosts to be occupied and the prevalence dropped to 35%. We may assume that chemically mediated cues of aggressiveness possibly released during the initial fighting could cause the loser symbiont not to risk entering a host in the same aquarium for a while. However, for a symbiont, the stimulus of finding and colonizing a host must be much compelling. Alternatively, we suggest that the winner symbiont did not allow the loser to enter any tube, even that of a second available hosts, being thus able to control not only its host, but the surrounding bottoms and nearby hosts.

We have no information to accept or discard the presence of chemical cues. However, territorial interactions also occurred inside the host when CRR = 1, while chasing and fighting events occurred outside the host (when CRR = 2 and CRR = 1), supporting the hypothesis of an extended territory. In contrast, the single known previous experimental study using sequential reduction of competitor-to-resource ratio in an aquarium, resulted in the immediate occupation of the added free hosts^12^. Neither previous experiences, nor any supposed chemical cue prevented the attempts of *A. pulchra* to occupy a host. Certainly, we cannot discard the influence of the artificial conditions used in our experiments, and clarifying this question will require further dedicated experimentation. Nevertheless, accepting an extended territory agrees with three of our field observations: (1) most often, only one of two close hosts harbored a symbiont (despite the prevalence exceeding 50%), (2) when there were two symbionts in two closely located hosts, one of them had recent severe injuries, and (3) denser populations tend to show a lower prevalence.

The overall low host densities in Nhatrang Bay may limit the movement of symbionts between hosts and thus reduce the frequency of intruders, resulting in lower costs associated with territorial defense^65^. However, extending the territory beyond the host increased predation risk and required additional energy to protect it from intruders. This raised the question of the possible benefits, which we suggest may be related with feeding strategies. We did not specifically address the trophic behavior in our experiments. However, our long-term observations supported the absence of direct host/symbiont trophic relationships. We did not register any attempt to steal food from the host filtration system, as reported for other symbiotic scaleworms^66^. We can also discard the plankton or organic matter entering the tube with inhalant current produced by the host pumping as the main food source for the symbiont, as we only registered a single attempt to snatch a small particle inside the host tube in more than 88 h. of observations. Scaleworms are widely considered to be carnivorous^66^. Given the powerful pharynx armed with large chitinous jaws, the quick movements and the facility of leaving the host of *O. pettiboneae*, we suggest that it may use the controlled territory outside the host to forage. In any case, its feeding mode and the relationships with the territory size are interesting topics and merit further study.

The high level of intraspecific aggressiveness, the strictly uniform 1:1 distribution, and the low density of the host population also raises the question of how *O. pettiboneae* reproduces. Previously, we assumed broadcast reproduction^61^,suggesting aggregating behavior to facilitate fertilization, which could be achieved either through reducing aggressiveness during spawning allowing to aggregate either inside or outside the host or through populations synchronizing spawning, as in non-symbiotic polychaetes^67,68^. Although the low density of the Vietnamese host populations renders the second as less probable, none of these strategies can be currently confirmed. Future studies should focus on exploring the reproductive behavior of this fascinating species.

### Behavioral complexity in *Ophthalmonoe pettiboneae*

The behavioral repertoire of *O. pettiboneae* consisted of relatively simple actions (i.e., host entering, synchronization with host reversing) and a complex territoriality that included several behavioral events (e.g., conspecific recognition, chasing and escaping, choosing a position to attack, single or several consecutive attacks, biting mainly around the pharyngeal area). Its order and frequency varied depending on the symbionts’ characteristics. For instance, a small symbiont trying to escape without entering into conflict, or a large intruder symbiont having suffered from an attack from a small resident can almost immediately respond by attacking its competitor. This suggests an ability to choose among competitive outcomes, which is a much more complex behavior than the simple biting when in contact with conspecifics described for other symbiotic polychaetes^12,43,49^. This behavior also differs significantly from the sequence of ritualized actions described for the intraspecific fights between crabs and crayfish^5,14,69,70^. Although some of these behavioral features are known for different species of symbiotic polychaetes and crabs, they have never been previously reported all together in a single polychaete species.

The territorial behavior of *O. pettiboneae* was also characterized by its aggressive fighting. We did not observed any traits of ritualized behavior aimed at reducing injury risks during an encounter, as in symbiotic crabs^5,14^. Only when the opponent’s size was clearly different, small worms avoided conflicts. Therefore, almost all agonistic interactions led to bites and injuries of parapodia or elytra in one or both contestants, likely being costly for both opponents, but especially for the loser that can even be torn in two parts. The attacks could even result in having the smaller symbiont divided in two fragments. This level of aggressiveness in intraspecific fighting has never been previously recorded in symbiotic polychaetes. The results of intraspecific encounters definitely depended on the relative size of the opponents. Large symbionts always won the intraspecific collisions observed and remained preferentially inside the host. Even if resident symbionts could have an advantage over the intruders, as in other invertebrates^65^, their relative size was also influencing. In fact, the only encounter involving a small resident led to especially intense interactions including three consecutive attacks and injuries to both competitors. In turn, the multiple injuries in most of the symbionts at the end of our experiments suggested more interactions than those we observed directly.

The lateral injuries we found in recently collected individuals were identical to those resulting from intraspecific fighting in our experiments. Thus, we provide the first empirical evidence on the previously hypothesized origin of injuries^47,48,71^. It was also hypothesized that posterior injuries may have different causes, including interspecific predation^47,48,71^. However, our results proved that these could also be caused by intraspecific aggression. During the attacks a symbiont could even result divided in two fragments. Although this appeared to be a harmful damage, it could be explained by autotomy (a typical escape reaction in many free-living polychaetes^72,73^) triggered by the attack.

Many more questions cannot be answered in light of our results. The lack of size-relationships between host and symbiont suggested lack of fidelity in the association of *O. pettiboneae* to a given host individual. However, host entering implied host species recognition, which may either be visual (i.e., through the tube siphons, at least in natural conditions), chemical (i.e., through the host exhalant current) or mechanical (by contacting with the siphon) or a combination of all them. Also, we cannot discount the fact that some intraspecific interactions (e.g., initial detection or chasing conspecifics) could be chemically mediated, as occur in social interactions in crayfish^69^, symbiotic crabs^6^ and polychaetes, including symbionts^18,37^. Nevertheless, some of the observed behavioral traits lead us to suggest a visual component: (1) a scaleworm approaching a conspecific only recognized and reacted to its presence after having the head oriented towards it, (2) the attacks always affected the anterior-most region of the victim, and (3) to prevent being perceived, the aggressor kept the host body in between when approaching to attack the intruder. This was consistent with the large, forward-facing pairs of anterior eyes with lenses. Among polynoids, this trait is exclusive of *O. pettiboneae* and can facilitate living inside tubes^59,61^, but also targeting objects. These objects could be conspecifics, but also preys. Thus, we hypothesize that *O. pettiboneae* may behave as an active predator with a hunting territory including the area surrounding the host tube.

*Ophthalmonoe pettiboneae* was undoubtedly a specialized symbiont, that seemed to be well-adapted to live inside chaetopterid hosts, but had never been found associated to other species than *C.* cf. *appendiculatus*, including the syntopic *Chaetopterus* sp.^46^. It could benefit from shelter and a continuous supply of fresh oxygenated water and, likely, food, while its presence neither damaged, nor benefited the host. Thus, we consider the association as an obligate commensalism, close to an inquilinism, as previously postulated for other symbiotic scaleworms^74^.

## Conclusions

The complex behavioral repertoire of *O. pettiboneae*, first reported for a polychaete, included territoriality, which (1) was expressed as intraspecific aggressive interactions, (2) led to host monopolization by one individual symbiont, and (3) seemed to favor a uniform 1:1 distribution in natural populations. Moreover, for the first time for a marine symbiotic invertebrate, our results suggest that *O. pettiboneae* may control a territory extending beyond the inner host tube to include adjacent bottoms and even neighboring hosts.

The evident aggressiveness in the territorial fighting of *O. pettiboneae* apparently lacked any ritualized behavior aiming at reducing the risk of receiving injuries, and our results provide the first empirical support that they were identical to those found in natural populations of symbiotic scaleworms. We thus conclude that injuries allow predicting intraspecific confrontations in symbiotic polychaetes.

The association between *O. pettiboneae* and *C.* cf. *appendiculatus* revealed to be an amazing model for further studies on the symbiotic associations involving marine invertebrates. Multiple interesting questions remain still open, including the function of the specialized eyes, the importance of autotomy and regeneration processes, and the relationships between a strict uniform distribution and reproduction and between territoriality and diet.

We finally conclude that *O. pettiboneae* behaved as a highly specialized symbiont that seemed to be perfectly adapted to live with its host. It received shelter, oxygen and, maybe, food supply, but we did not find evidence of host damages, nor of benefits. We thus consider the association as an obligate commensalism, close to an inquilinism.

## Material and methods

### Sampling and in situ host density assessment

We conducted sampling from March to April 2016 in four localities of Nhatrang Bay (Vietnam, South China Sea): the western coast of Mun Island (12° 10′ 10″ N, 109° 17′ 46″ E, 13–16 m depth), the southern coast of Mot Island (12° 10′ 26″ N, 109° 16′ 23″ E, 16–20 m depth), Point Nam at the western coast of Tre Island (12° 13′ 42″ N, 109° 13′ 47″ E, 10–12 m depth) and Dam Bay (12° 11′ 43″ N, 109° 17′ 26″ E, 6–8 m depth).

We collected all organisms used in the laboratory haphazardly in all sampling locations by SCUBA diving, under the official permission of the Russian-Vietnamese Tropical Center. The U-shaped tubes of *Chaetopterus* cf. *appendiculatus* (Supplementary material S1) were detected underwater by the siphons, which emerged about 1–5 cm from the sediment surface (Supplementary material S1), while the hidden portion of the tube was buried about 15–20 cm deep in the sediment. Extraction was achieved by blocking the two tube siphons with our fingers (to prevent worms from escaping) and then gently washing out the sediments over the tube by hand venting, until the whole tube was exposed (Supplementary material S2). The tubes were then carefully removed, immediately placed into individual zip-lock plastic bags, transferred to seawater tanks and transported to the laboratory.

Based on visually detecting tube siphon pairs, we estimated the density of *C.* cf. *appendiculatus* as number of worms seen per diving hour at each sampling site. One diver was swimming along isobaths at approximately 0.3 km h^−1^ to annotate each record. Complementary counting (in different days and only at Mun Island and Dam Bay) were performed along five 50 m long parallel transects following the depth profile (each one immediately adjacent to the previous one but ~ 2 m apart) by two divers (each one on 1 m at each side). Densities were expressed as ind.·500 m^−2^.

### Sample processing and maintenance of organisms

Seawater used in the laboratory was obtained from Nhatrang Bay, close to the locations where the worms were collected and kept aerated in a refrigerated laboratory (28–30 °C, the in situ recorded temperature) for at least 24 h. before starting any procedure. Other variables were not measured. Oxygenation was assured by a continuous aeration system and we assume there were no changes in salinity during the transport from the field to the laboratory.

In the laboratory, we first measured the host tube length to the nearest 5.0 mm. Then, we carefully cut each tube along its longitudinal axis with the help of round tipped scissors to collect and count the chaetopterids and, when present, the scaleworms, to estimate the infestation prevalence (as percentage of infested vs. total hosts) for each study site. We used seawater to pour each symbiont from the host tube into individual labeled Petri dishes. All scaleworms were photographed, measured to the nearest 0.1 mm (as body length from tip of prostomium to the end of pygidium) and checked for injuries, either lateral (on parapodia or elytra) or posterior (lacking whole most posterior region). We avoided handling the delicate chaetopterids as much as possible, including trying relaxing them. Measuring the length of living individuals was extremely difficult because its continuous elongation and contraction, even leading to fragmentation. Therefore, we poured each host directly from the opened tube to a 200 ml graduated vessel half filled with seawater and used volume (as displaced seawater measured to the nearest 1 ml) as size proxy. We analyzed the relationships between symbiont length and host tube length and body volume by linear regression analyses using the XLSTAT software version 18.03 by Addinsoft 1995–2017.

**Figure 8:**
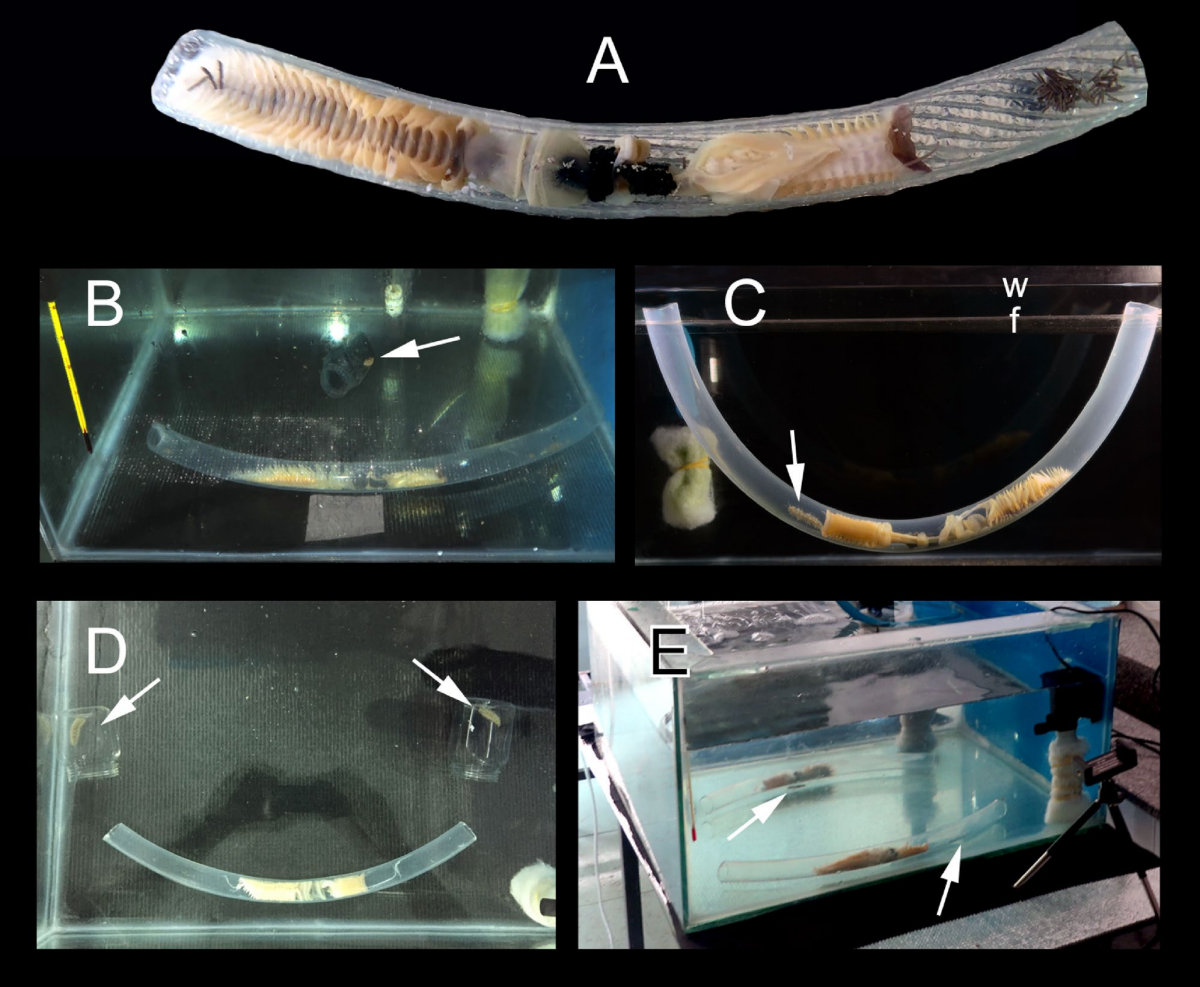
(**A**) *Chaetopterus* cf. *appendiculatus* inside a transparent plastic tube. (**B**) Experimental aquarium with one host in a plastic tube and one symbiont. (**C**) Experimental aquarium with perforated false bottom and a fixed plastic tube containing one host and two symbionts. (**D**) Manipulating competitor-to-resource ratio (CRR) experiment, CRR = 2 treatment (one host, two symbionts)*.* (**E**) CRR manipulative experiments, CRR = 1 treatment (two host, two symbionts)*.* White arrows pointing on symbionts’ position.

*Chaetopterus* and the associated symbionts can be extracted from the original host tube and transferred to transparent artificial tubes, allowing direct observations of their behavior in laboratory conditions^63^. Therefore, we poured each host from the graduate vessel to a transparent plastic tube of 25 mm in diameter, equivalent to the original parchment-like tube (Fig. 8A). Before starting the experiments, each host inside a plastic tube and each symbiont inside a perforated plastic jar were placed at the bottom of individual acclimatization aquaria with running seawater for at least 24 h.

### Experimental set-ups

We carry out our observations (88 h) either with the naked eye or by digital recordings (photos and high-definition videos) obtained with a digital camera Nikon D750 or an iPhone 8plus. The processed images and videos are included in the corresponding figures and provided as supplementary materials, respectively.

#### Host/symbiont experiment

This experiment was performed to describe the symbiont behavior during its entrance into the host. We transferred one host inside its plastic tube from the acclimatization aquarium to an experimental aquarium (50 cm long by 50 cm wide by 30 cm high), let it acclimatize for 2 h. Then we also transferred one symbiont in a plastic jar from its own acclimatization aquarium, placing the opened jar on the bottom, with the opening facing, and close to, the host tube (Fig. 8B). Hosts and symbionts were used only once. We ran five 1 h. independent replicates and we recorded the symbiont host-entering behavior, its location inside the host tube after entering, and its behavior during host reversal movements by direct observations and continuous digital videos (3 h.). Complementary behavioral observations were obtained from the next two experimental set-ups.

#### Symbiont/symbiont experiment

This experiment was designed to obtain preliminary data on the influence of symbionts size and residence on the results of the agonistic encounters. We used a specially designed aquarium (40 cm long by 30 wide by 35 cm high) with a plastic plate perforated with two holes as a false bottom placed in its upper part. In these holes, we fixed a U-shaped plastic tube with one host and its symbiont (the resident), so that the tube remained in a vertical position (Fig. 8C). After 24 h. of acclimatization, we closed the open circulation system (keeping the seawater aerated) and placed another symbiont (the intruder) on the false bottom close to a tube opening. Observations ended when one of the symbionts left the host tube, and extended from 1 to 6 h. We established two treatments (one replicate each): (1) resident ≈ 5 mm longer than intruder and (2) intruder ≈ 5 mm longer than resident. We registered them using a digital camera with a time lapse (one photo each 15 s).

#### Manipulating competitor-to-resource ratio (CRR) experiment

This experiment was designed to assess whether the agonistic behavior was at the origin of the establishment of the symbiont’s uniform distribution. Treatments consisted on sequentially decreasing the competitor-to-resource ratio, from two (CRR = 2, two symbionts/one host) to one (CRR = 1, two symbionts/two hosts), assuming that the symbiont remaining outside when CRR = 2 would occupy the newly added host. In each replicate, symbionts differed ca. 5 mm in size and were from hosts different to those in the replicate. Hosts and symbionts were used only once.

All replicates were conducted in 50 cm long by 50 cm wide by 30 cm high aquaria with close circulation and aerated seawater. In the CRR = 2 treatment (15 replicates), two plastic jars with scaleworms were placed in the aquaria lying on one side, with the opening facing the openings of the host tube (Fig. 8D). Each replicate started at ca. 2000–2100 and extended for 24 h. Then, we registered the positions of the two symbionts, partially renewed seawater, introduced an additional host (Fig. 8E) and registered again the positions of the two symbionts after 24 h. CRR = 1 treatment had only 13 replicates (after the first treatment, one symbiont was too fragmented and it was not possible to find another one). During each treatment, we observed the symbionts behavior every 3 h. during 10–90 min (depending on their activity), excluding night time (1200–0600), but only their final positions were considered in the statistical analysis. Before and after each replicate, we checked each scaleworm for the presence of lateral and posterior injuries.

We expected four possible scenarios: 1 (each host harboring one symbiont), 2 (two symbionts sharing one host), 3 (both symbionts outside), and 4 (only in the CRR = 1 treatment, one symbiont inside a host and the other outside). We assumed that a significantly higher frequency of scenario 1 would confirm the intraspecific agonistic interactions as leading to the establishment of a 1:1 uniform distribution, of scenario 2 would indicate low (or null) intraspecific antagonism, and of scenarios 3 and 4 would indicate the existence of interactions outside the host, with scenario 4 even supporting that the territory could not be limited to a single host. We checked whether the observed distributions differed from expected at random with non-parametric contingency tables generated by Montecarlo simulations (5,000) having the same marginal sums as the observed data. We computed a chi-square statistic for each simulated table and the corresponding *p* values by calculating the distribution obtained from the simulations. We then estimated the contribution of each table cell to the chi-square, as well as the significance (*p* ≤ 0.05, Fisher’s exact test) of the differences between observed and simulated values^75,76^. These analyses were performed with the XLSTAT software version 18.03 by Addinsoft 1995–2017.

## Supplementary Information

Supplementary Information.

## Data Availability

The data sets analyzed during the current study are available from TAB on reasonable request.

## References

[1] Baeza JA, Thiel M (2003). Predicting territorial behavior in symbiotic crabs using host characteristics: A comparative study and proposal of a model. Mar. Biol..

[2] Kamran M, Moore PA, Shackelford TK, Weekes-Shackelford VA (2016). Dominance and territory. Encyclopedia of Evolutionary Psychological Science.

[3] Grant JWA (1993). Whether or not to defend? The influence of resource distribution. Mar. Behav. Physiol..

[4] Duffy JE, Kikuchi T (2002). The ecology and evolution of eusociality in sponge-dwelling shrimp. Genes, Behaviors and Evolution of Social Insects.

[5] Baeza JA, Stotz W, Thiel M (2002). Agonistic behaviour and development of territoriality during ontogeny of the sea anemone dwelling crab *Allopetrolisthes spinifrons* (H. Milne Edwards, 1837)(Decapoda: Anomura: Porcellanidae). Mar. Freshw. Behav. Physiol..

[6] Castro P, Castro P (2015). Symbiotic Brachyura. Treatise on Zoology-Anatomy, Taxonomy, Biology. The Crustacea, Volume 9 Part C.

[7] Wilson EO (1975). Sociobiology: The New Synthesis.

[8] Burt WH (1943). Territoriality and home range concepts as applied to mammals. J. Mammal..

[9] Gerking SD (2014). Feeding Ecology of Fish.

[10] Barrows EM (2000). Animal Behavior Desk Reference: A Dictionary of Animal Behavior, Ecology, and Evolution.

[11] Hardy ICW, Briffa M (2013). Animal Contests.

[12] Dimock RV, Vernberg WB (1974). Intraspecific aggression and the distribution of a symbiotic polychaete on its host. Symbiosis in the Sea.

[13] Duffy JE, Morrison CL, Macdonald KS (2002). Colony defense and behavioral differentiation in the eusocial shrimp *Synalpheus regalis*. Behav. Ecol. Sociobiol..

[14] Huber ME (1987). Aggressive behavior of Trapezia intermedia Miers and T. digitalis Latreille (Brachyura: Xanthidae). J. Crustacean Biol..

[15] Douglas A (2010). The Symbiotic Habit.

[16] Williams JD, McDermott JJ (2004). Hermit crab biocoenoses: A worldwide review of the diversity and natural history of hermit crab associates. J. Exp. Mar. Biol. Ecol..

[17] Fautin DG (1991). The anemonefish symbiosis: What is known and what is not. Symbiosis.

[18] Martin D, Britayev TA (1998). Symbiotic polychaetes: Review of known species. Oceanogr. Mar. Biol. Ann. Rev..

[19] Fernández-Leborans G (2010). Epibiosis in Crustacea: An overview. Crustaceana.

[20] Stella JS, Pratchett MS, Hutchings PA, Jones GP (2011). Diversity, importance and vulnerability of coral-associated invertebrates. Oceanogr. Mar. Biol. Ann. Rev..

[21] Thiel M, Baeza JA (2001). Factors affecting the social behaviour of crustaceans living symbiotically with other marine invertebrates: a modelling approach. Symbiosis.

[22] Jones KMM (2005). The effect of territorial damselfish (family Pomacentridae) on the space use and behaviour of the coral reef fish *Halichoeres bivittatus* (Bloch, 1791) (family Labridae). J. Exp. Mar. Biol. Ecol..

[23] Thiel M, Zander A, Baeza JA (2003). Movements of the symbiotic crab *Liopetrolisthes mitra* between its host sea urchin *Tetrapygus niger*. Bull. Mar. Sci..

[24] Marin I, Britayev TA (2014). Symbiotic Community Associated with Corals Galaxea Oken, 1815 (Euphillidae: Scleractinia).

[25] Ross RM (1978). Territorial behavior and ecology of the anemonefish *Amphiprion melanopus* on Guam. Z. Tierpsychol..

[26] Kobayashi M, Hattori A (2006). Spacing pattern and body size composition of the protandrous anemonefish *Amphiprion frenatus* inhabiting colonial host anemones. Ichthyol. Res..

[27] Huebner LK, Dailey B, Titus BM, Khalaf M, Chadwick NE (2012). Host preference and habitat segregation among Red Sea anemonefish: Effects of sea anemone traits and fish life stages. Mar. Ecol. Progr. Ser..

[28] Duffy JE (1996). Eusociality in a coral-reef shrimp. Nature.

[29] Baeza JA, Stotz WB (2001). Host-use pattern and host-selection during ontogeny of the commensal crab *Allopetrolisthes spinifrons* (H. Milne Edwards, 1837) (Decapoda: Anomura: Porcellanidae). J. Nat. Hist..

[30] Ambrosio LJ, Baeza JA (2016). Territoriality and conflict avoidance explain asociality (solitariness) of the endosymbiotic pea crab *Tunicotheres moseri*. PLoS ONE.

[31] Baeza JA, Thiel M, Duffy JE, Thiel M (2007). The mating system of symbiotic crustaceans: A conceptual model based on optimality and ecological constraints. Evolutionary Ecology of Social and Sexual Systems: Crustaceans as Model Organisms.

[32] Bell JL (1988). Distribution and abundance of *Dissodactylus mellitae* Rathbun (Pinnotheridae) on *Mellita quinquiesperforata* (Leske)(Echinodermata). J. Exp. Mar. Biol. Ecol..

[33] Castro P (1978). Movements between coral colonies in *Trapezia ferruginea* (Crustacea: Brachyura), an obligate symbiont of scleractinian corals. Mar. Biol..

[34] Baeza JA, Simpson L, Ambrosio LJ, Guéron R, Mora N (2016). Monogamy in a hyper-symbiotic shrimp. PLoS ONE.

[35] Diesel R (1988). Male-female association in the spider crab *Inachus phalangium*: The influence of female reproductive stage and size. J. Crustac. Biol..

[36] Wells HW, Wells MJ (1961). Observations on *Pinnaxodes floridensis*, a new species of pinnotherid crustacean commensal in holothurians. Bull. Mar. Sci..

[37] Martin D, Britayev TA (2018). Symbiotic polychaetes revisited: an update of the known species and relationships (1998–2017). Oceanogr. Mar. Biol. Ann. Rev..

[38] Perry O, Sapir Y, Perry G, Ten Hove H, Fine M (2018). Substrate selection of Christmas tree worms (*Spirobranchus* spp.) in the Gulf of Eilat, Red Sea. J. Mar. Biol. Ass. UK.

[39] Hunte W, Colin BE, Marsden JR (1990). Habitat selection in the tropical polychaete *Spirobranchus giganteus* 1 Distribution on corals. Mar. Biol..

[40] Mackie ASY, Oliver PG, Nygren A (2015). *Antonbruunia sociabilis* sp. nov (Annelida: Antonbruunidae) associated with the chemosynthetic deep-sea bivalve *Thyasira scotiae* Oliver & Drewery, 2014, and a re-examination of the systematic affinities of Antonbruunidae. Zootaxa.

[41] Ruff, R. E. A new species of *Bathynoe* (Polychaeta: Polynoidae) from the Northeast Pacific Ocean commensal with two species of deep-water asteroids. in: *Systematics, Biology and Morphology of World Polychaeta. Proceedings of the Second International Polychaeta Conference*. *Ophelia* **Suppl. 5**, 219–230 (1991).

[42] Miura T, Ohta S (1991). Two polychaete species from the deep-sea hydrothermal vent in the Middle Okinawa Trough. Zool. Sci..

[43] Martin D, Nygren A, Hjelmstedt P, Drake P, Gil J (2015). On the enigmatic symbiotic polychaete “*Parasyllidea*” *humesi* Pettibone, 1961 (Hesionidae): taxonomy, phylogeny and behaviour. Zool. J. Linn. Soc..

[44] Chim CK, Ong JJL, Tan KS (2013). An association between a hesionid polychaete and temnopleurid echinoids from Singapore. Cah. Biol. Mar..

[45] Goerke H (1971). *Nereis fucata* (Polychaeta, Nereidae) als kommensale von *Eupagurus bernhardus* (Crustacea, Decapoda) Entwicklung einer population und verhalten der art. Veröffentlichungen des Instituts für Meeresforschung in Bremerhaven.

[46] Britayev TA, Mekhova E, Deart Y, Martin D (2017). Do syntopic host species harbour similar symbiotic communities? The case of *Chaetopterus* spp. (Annelida: Chaetopteridae). PeerJ.

[47] Britayev TA, Martin D, Krylova EM, von Cosel R, Aksiuk ES (2007). Life-history traits of the symbiotic scale-worm *Branchipolynoe seepensis* and its relationships with host mussels of the genus *Bathymodiolus* from hydrothermal vents. Mar. Ecol. Evolut. Perspect..

[48] Britayev TA, Zamyshliak EA (1996). Association of the commensal scaleworm *Gastrolepidia clavigera* (Polychaeta: Polynoidae) with holothurians near the coast of South Vietnam. Ophelia.

[49] Britayev, T. A. Life cycle of the symbiotic scale-worm *Arctonoe vittata* (Polychaeta: Polynoidae). In: *Systematics, Biology and Morphology of World Polychaeta. Proceedings of the Second International Polychaeta Conference. Ophelia* **Suppl. 5**, 305–312 (1991).

[50] Devaney DM (1967). An ectocommensal polynoid associated with Indo-pacific echinoderms, primarily ophiuroids. Occ. Pap. Bernice P. Bishop Mus..

[51] Tokaji H, Nakahara K, Goshima S (2014). Host switching improves survival rate of the symbiotic polychaete *Arctonoe vittata*. Plank. Bent. Res..

[52] Martin D, Rosell D, Uriz MJ (1992). *Harmothoe hyalonemae* sp. nov. (Polychaeta, Polynoidae), an exclusive inhabitant of different Atlanto-Mediterranean species of Hyalonema (Porifera, Hexactinellida). Ophelia.

[53] Reish DJ, Alosi MC (1968). Aggressive behavior in the polychaetous annelid family Nereidae. Bull. South. Calif. Acad. Sci..

[54] Evans SM (1971). Behavior in polychaetes. Q. Rev. Biol..

[55] Scaps P (1995). Intraspecific agonistic behaviour in the polychaete *Perinereis cultrifera* (Grübe). Vie et Milieu.

[56] Johnson HP (1897). A preliminary account of the marine annelids of the Pacific coast, with descriptions of new species. Proc. Calif. Acad. Sci..

[57] Miers, E. J. Report on the Brachyura collected by HMS Challenger during the years 1873–1876. in: Report on the scientific results of the Voyage of HMS Challenger during the years 1873–76 under the command of Captain George S. Nares, R. N., F.R.S. and the late Captain Frank Tourle Thompson, R. N. *Zoology* **17**, 1–363, pls. 361–329 (1886).

[58] Latreille, P. A. Trapezie. in *Entomologie, ou histoire naturelle des crustaces, des arachnides et des insectes*, Vol. 10 695–696 (Encyclopedie Methodique, Histoire Naturelle, 1828).

[59] Petersen ME, Britayev TA (1997). A new genus and species of polynoid scaleworm commensal with *Chaetopterus appendiculatus* Grube from the Banda Sea (Annelida: Polychaeta), with a review of commensals of Chaetopteridae. Bull. Mar. Sci..

[60] Grube AE (1874). Descriptiones Annulatorum novorum mare Ceylonicum habitantium ab honoratissimo Holdsworth collectorum. Proc. Zool. Soc. Lond..

[61] Britayev TA, Martin D (2005). Scale-worms (Polychaeta, Polynoidae) associated with chaetopterid worms (Polychaeta, Chaetopteridae), with description of a new genus and species. J. Nat. Hist..

[62] Grant JWA, Gaboury CL, Levitt HL (2000). Competitor-to-resource ratio, a general formulation of operational sex ratio, as a predictor of competitive aggression in Japanese medaka (Pisces: Oryziidae). Behav. Ecol..

[63] Britayev TA, Smurov AV (1988). Distribution and relocation of commensal crabs *Pinnixa rathbhuni* (Pinnotheridae) on their hosts. Dokl. Akad. Nauk SSSR.

[64] Walker AO (1887). Notes on a collection of Crustacea from Singapore. J. Linn. Soc. Lond. Zool..

[65] Kemp DJ, Córdoba-Aguilar A, González-Tokman D, González SI (2018). Habitat selection and territoriality. Insect behavior: from mechanisms to ecological and evolutionary consequences.

[66] Jumars PA, Dorgan KM, Lindsay SM (2015). Diet of worms emended: An update of polychaete feeding guilds. Ann. Rev. Mar. Sci..

[67] Cotter E, O'Riordan RM, Myers AA (2003). A histological study of reproduction in the serpulids *Pomatoceros triqueter* and *Pomatoceros lamarckii* (Annelida: Polychaeta). Mar. Biol..

[68] Prevedelli D, Massamba N’Siala G, Ansaloni I, Simonini R (2007). Life cycle of *Marphysa sanguinea* (Polychaeta: Eunicidae) in the Venice Lagoon (Italy). Mar. Ecol..

[69] Bergman DA, Moore PA (2005). Prolonged exposure to social odours alters subsequent social interactions in crayfish (*Orconectes rusticus*). Anim. Behav..

[70] Arakaki JY (2020). Battle of the borders: Is a range-extending fiddler crab affecting the spatial niche of a congener species?. J. Exp. Mar. Biol. Ecol..

[71] Britayev TA, Mekhova ES (2014). Do symbiotic polychaetes migrate from host to host?. Mem. Mus. Victoria.

[72] Livermore J, Perreault T, Rivers T (2018). Luminescent defensive behaviors of polynoid polychaete worms to natural predators. Mar. Biol..

[73] Daly JM (1973). Segmentation, autotomy and regeneration of lost posterior segments in *Harmothoe imbricata* (L) (Polychaeta: Polynoidae). QH1.M454.

[74] Schiaparelli S, Alvaro MC, Barnich R (2011). Polynoid polychaetes living in the gut of irregular sea urchins: A first case of inquilinism in the Southern Ocean. Antarct. Sci..

[75] Sokal RR, Rohlf FJ (1995). Biometry. The Principles and Practice of Statistics in Biological Research.

[76] Everitt B (1992). The Analysis of Contingency Tables.

