# Supplementary material for: Behavioral traits and territoriality in the symbiotic scaleworm Ophthalmonoe pettiboneae
Source: Sci Rep. 2021 Jun 11;11:12408. doi: 10.1038/s41598-021-91810-2 (PMC8195992; doi:10.1038/s41598-021-91810-2)
Supplement: Supplementary file 1 — Supplementary Information. [file 41598_2021_91810_MOESM1_ESM.pdf]

Supplementary material S1

Main characteristics of the host *Chaetopterus* cf. *appendiculatus* Grube, 1874 [1] (Annelida: Chaetopteridae) and its symbiont *Ophthalmonoe pettiboneae* Petersen and Britayev, 1997 [2] (Annelida: Polynoidae).

The host

Chaetopterus cf. *appendiculatus* is an undescribed species that may grow up to be 20 cm long and lives in U-shaped, parchment-like tubes buried in subtidal (6–20 m depth) soft sediments (Fig. S1A–B). At present, it is only known from Nhatrang Bay in Vietnam [3]. Its tube-dwelling behavior creates a well-protected shelter, likely continuously providing oxygen and food (e.g., plankton and sediment particles) for three specialized symbionts: *O. pettiboneae*, more rarely, the porcellanid crab *Eulinaios cometes* (Walker, 1887) [4] and the pearlfish *Onuxodon fowleri* (Smith, 1964) [5]. Only the first two, and only once, were observed to share the same host individual [3].

The body of *C.* cf. *appendiculatus* consists of three regions: A (nine anterior-most segments), B (three mid-body segments), and C (tenths of posterior-most segments) (Fig. S1C). Like other species of the genus, it is adapted to feed on plankton using a mucus net coupled with pumping water by the synchronized beating of the fan-like region B segments [6-8]. The mucus net is placed between the tips of the aliform notopodia and the feeding organ or cupule (both in the first parapodia of region B) (Fig. S1C) [9]. Water currents are also used to expel feces and irritating particles by inverting the beating segments. The two siphons are alternatively used as inhalant and exhalant, as the worms can turn around very rapidly in the tube causing a change in current direction (Supplementary material S12).

Since body reversing may influence the behavior of the symbiont, we are here providing a detailed description. The chaetopterid lay with the ventral side in contact with the lower tube wall, regularly pumping water. After pumping stopped, body reversing start with a slight retraction of the anterior end, followed by bending up the lip and then the whole region A. The plastron (i.e., ventral side of region A) is detached from the lower inner tube wall and then attached to the upper one. The whole anterior body (including region B) moves back, turning along its longitudinal axis so that its ventral side faces the upper tube wall and then remains almost static. Finally, region C keep moving back to quickly complete reversing and

the worm starts again pumping water. Typically, the whole sequence extends for 1.5 to 2 minutes.

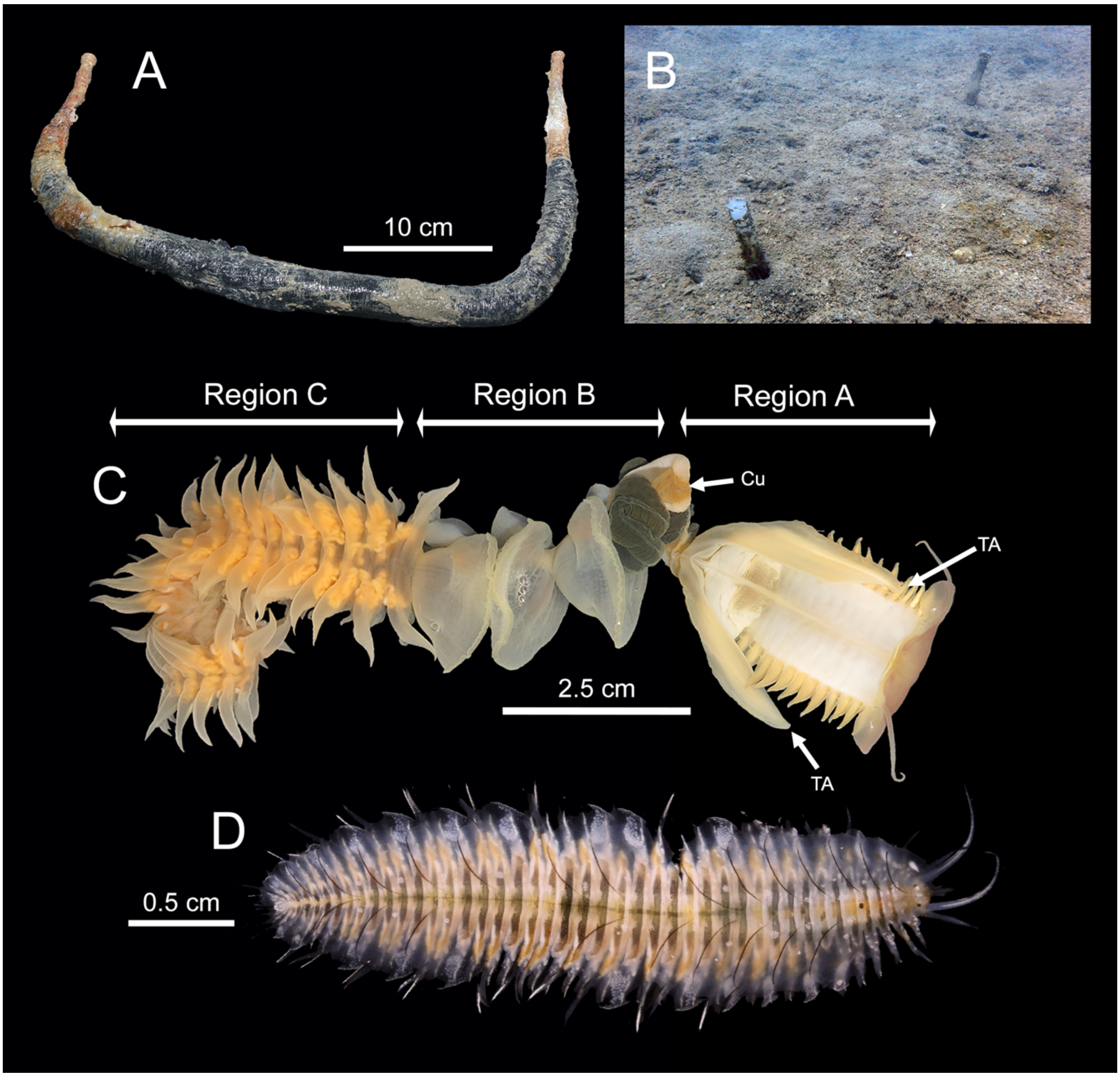

Fig. S1. A–C. *Chaetopterus* cf. *appendiculatus*. A. Tube extracted from sediment. B. Tube *in situ*, showing the two siphons protruding from the sediment. C. A specimen extracted from the tube, showing the three body regions and the location of the filtration bag between the tips of the aliform notopodia (TA) and the cupule (Cu). D. *Ophthalmonoe pettiboneae*. Whole body in dorsal view. B, C: modified from Britayev et al. [3].

The symbiont

Ophthalmonoe pettiboneae was first described based on a specimen from the Banda Sea (Indonesia) and later redescribed based on specimens from Vietnam [2,10]. It may reach up to 41 mm long and has a flat, overall white colored body, with a large blackish central band and

one central and two lateral orange, spots on each segment. The body is covered with large, hyaline elytra with a tiny black line on the posterior border and long dorsal cirri, which are colored an alternating black and white (Fig. S1D). It differs from other polynoids in having a very large pair of anterior eyes, placed on the anterior prostomial edge and directed frontally, which has been considered as an adaptation to living inside the tube of its chaetopterid host [2,10].

References

1 Grube, A. E. Descriptiones Annulatorum novorum mare Ceylonicum habitantium ab honoratissimo Holdsworth collectorum. *Proc. Zool. Soc. London* **41**, 325-329, doi:https://doi.org/10.1111/j.1096-3642.1874.tb02492.x (1874).

2 Petersen, M. E. & Britayev, T. A. A new genus and species of polynoid scaleworm commensal with *Chaetopterus appendiculatus* Grube from the Banda Sea (Annelida: Polychaeta), with a review of commensals of Chaetopteridae. *Bull. Mar. Sci.* **60**, 261-276 (1997).

3 Britayev, T. A., Mekhova, E., Deart, Y. & Martin, D. Do syntopic host species harbour similar symbiotic communities? The case of *Chaetopterus* spp. (Annelida: Chaetopteridae). *PeerJ* **5**, e2930, doi:https://doi.org/10.7717/peerj.2930 (2017).

4 Walker, A. O. Notes on a collection of Crustacea from Singapore. *Journal of the Linnean Society of London, Zoology* **20**, 107-117, doi:https://doi.org/10.1111/j.1096-3642.1887.tb01440.x (1887).

5 Smith, C. L. Some pearlfishes from Guam, with notes on their ecology. *Pac. Sci.* **18**, 34-40 (1964).

6 Enders, H. E. A study of the life-history and habits of *Chaetopterus variopedatus*. *J. Morph.* **20**, 479-531, doi:https://doi.org/10.1002/jmor.1050200306 (1909).

7 MacGinitie, G. E. The method of feeding of *Chaetopterus*. *Biological Bulletin Marine Biological Laboratory, Woods Hole* **77**, 115-118, doi:https://doi.org/10.2307/1537850 (1939).

8 Wells, G. P. & Dales, R. P. Spontaneous activity patterns in animal behaviour: the irrigation of the burrow in the polychaetes *Chaetopterus variopedatus* Renier and *Nereis diversicolor* O. F. Müller. *J. Mar. Biol. Ass. UK* **29**, 661-680, doi:https://doi.org/10.1017/S0025315400052851 (1951).

9 Britayev, T. A. & Martin, D. in *Volume 1: Annelida basal groups and Pleistoannelida, Sedentaria; in: Purschke, G., Böggermann, M., Westheide, W. (Eds.), Annelida; in: Schmidt-Rhaesa, A. (Ed.), Handbook of Zoology* (eds G. Purschke, M. Böggermann, & W. Westheide) 156-174 (De Gruyter, 2019).

10 Britayev, T. A. & Martin, D. Scale-worms (Polychaeta, Polynoidae) associated with chaetopterid worms (Polychaeta, Chaetopteridae), with description of a new genus and species. *J. Nat. Hist.* **39**, 4081-4099, doi:https://doi.org/10.1080/00222930600556229 (2005).

Supplementary material S2

Hand-venting of sediments to collect a tube of *Chaetopterus* cf. *appendiculatus*. https://saco.csic.es/index.php/s/FkfKM582qbNAHe7

Supplementary material S3

Host entering. https://saco.csic.es/index.php/s/4X7kTN6GHbaJPpX

Supplementary material S4

Host venting and preferred symbiont location. https://saco.csic.es/index.php/s/6Dz3nwMRo9C7Q33

Supplementary material S5

Synchronized host/symbiont behavior. https://saco.csic.es/index.php/s/gWC4din6ktfqnpF

Supplementary material S6

Territorial interactions between a large resident and a small intruder inside the host tube. https://saco.csic.es/index.php/s/tMQPCeQA3ZyXd8J

Supplementary material S7

Territorial interactions between a small resident and a large intruder inside the host tube, with the latter revealing to be dominant. https://saco.csic.es/index.php/s/8y2kA74g3e44dyW

Supplementary material S8

Territorial interactions between large and small symbionts outside the host tube, with the former chasing the latter. https://saco.csic.es/index.php/s/iSYjY8JnMDe8Z83

Supplementary material S9

Territorial interactions between large and small symbionts outside the host tube, with the former successively attacking the latter. https://saco.csic.es/index.php/s/s7SneRf6KxAXbw8

Supplementary material S10

First example of scape reaction outside the tube. https://saco.csic.es/index.php/s/n9BdjqL2ByFrjYB

Supplementary material S11

Second example of scape reaction outside the tube.
https://saco.csic.es/index.php/s/qJiKDFSmrA88sRj

Supplementary material S12

Chaetopterus cf. *appendiculatus* reversing inside the tube.
https://saco.csic.es/index.php/s/WxDqdnBoRHC7wf3
